# 

**Published:** 1876-08

**Authors:** 


ESTABLISHED IN 1855.

VOLUME XIV. | AUGUST—1876. | NUMBER 5.

### ATLANTA

### Medical and Surgical Journal.

INDEXED
S.G.O.L.

MONTHLY: THREE DOLLARS PER ANNUM, IN ADVANCE.

EDITORS:

W. F. WESTMORELAND, M.D.

*Professor Principles and Practice of Surgery in Atlanta Medical College.*

AND

ROBERT BATTEY, M.D.

W. S. KENDRICK, M.D.,

*Associate Editor and Business Manager.*

*PAX ET SCIENTIA, SED VERITAS SINE TIMORE.*

ATLANTA, GEORGIA:

*DUNLOP & DICKSON, BOOK AND JOB PRINTERS.*

1876.

